# Evaluation of Brain Iron Content Based on Magnetic Resonance Imaging (MRI): Comparison among Phase Value, R2* and Magnitude Signal Intensity

**DOI:** 10.1371/journal.pone.0031748

**Published:** 2012-02-20

**Authors:** Shen-Qiang Yan, Jian-Zhong Sun, Yu-Qing Yan, He Wang, Min Lou

**Affiliations:** 1 Department of Neurology, the 2nd Affiliated Hospital of Zhejiang University, School of Medicine, Hangzhou, People's Republic of China; 2 Department of Radiology, the 2nd Affiliated Hospital of Zhejiang University, School of Medicine, Hangzhou, People's Republic of China; 3 Global Applied Science Laboratory, GE Healthcare, Shanghai, People's Republic of China; The Mental Health Research Institute of Victoria, The University of Melbourne, Australia

## Abstract

**Background and Purpose:**

Several magnetic resonance imaging (MRI) techniques are being exploited to measure brain iron levels increasingly as iron deposition has been implicated in some neurodegenerative diseases. However, there remains no unified evaluation of these methods as postmortem measurement isn't commonly available as the reference standard. The purpose of this study was to make a comparison among these methods and try to find a new index of brain iron.

**Methods:**

We measured both phase values and R2* in twenty-four adults, and performed correlation analysis among the two methods and the previously published iron concentrations. We also proposed a new method using magnitude signal intensity and compared it with R2* and brain iron.

**Results:**

We found phase value correlated with R2* in substantia nigra (r = −0.723, p<0.001) and putamen (r = −0.514, p = 0.010), while no correlations in red nucleus (r = −0.236, p = 0.268) and globus pallidus (r = −0.111, p = 0.605). And the new magnitude method had significant correlations in red nucleus (r = −0.593, p = 0.002), substantia nigra (r = −0.521, p = 0.009), globus pallidus (r = −0.750, p<0.001) and putamen (r = −0.547, p = 0.006) with R2*. A strong inverse correlation was also found between the new magnitude method and previously published iron concentrations in seven brain regions (r = −0.982, P<0.001).

**Conclusions:**

Our study indicates that phase value may not be used for assessing the iron content in some brain regions especially globus pallidus. The new magnitude method is highly consistent with R2* especially in globus pallidus, and we assume that this approach may be acceptable as an index of iron content in iron-rich brain regions.

## Introduction

Iron plays important roles in many biological processes ranging from facilitating cellular aerobic metabolism to participating in neurotransmitter synthesis and myelin production [1]–[4]. However, if iron is not properly regulated, it can be detrimental to neurons and contributes to the pathogenesis of many neurological diseases. Abnormal brain iron accumulation has been reported in many neurodegenerative diseases including Parkinson's disease, multiple systems atrophy, Huntington's disease, Alzheimer diseases, neuroferritinopathy and Hallervorden–Spatz disease and suggests that iron burden contributes to age- and disease-related functional decline, which thus represents potent targets for pharmacological manipulation to limit the progression of those diseases [5]–[10].

Moreover, iron content in the brain and hematoma are causally related to delayed neuronal injury and edema formation after intracranial hemorrhage [11]–[14]. Therefore, it is believed that the ability to quantitatively assess regional brain iron has a potential role in the diagnosis of disease, as well as understanding pathogenesis, disease progression and the monitoring of treatment.

There has been considerable interest in the use of MR imaging to assess iron concentration in the human brain over the last two decades. Typically, the high-iron regions have a hypointense (dark) signature on T2-weighted MR images and the presence of iron also leads to signal changes in T2*-weighted gradient echo images. The field-dependent rate increase (FDRI) technique, which measures the difference in R2 at 1.5 and 0.5 T was found to correlate strongly with iron concentration in healthy adults [15].

More recently, improved MRI techniques, such as susceptibility-weighted imaging (SWI) [16], [17], is potentially useful for the study of diseases with abnormal iron distributions. Several previous studies have demonstrated correlations between SWI phase shifts and brain iron concentration in different brain regions of healthy adults [18]–[20]. A postmortem validation study [21] also has showed a linear correlation between R2* and brain iron concentration. However, quantitative studies to verify the relationship between R2*, phase values of SWI and brain-iron concentration have not universally agreed on the strength of the correlation. In two reports, phase-sensitive methods did not correlate with iron content, compared with R2 * [22] or FDRI technique [20]. Moreover, there is no report on the relationship between iron concentration and magnitude SWI dataset, which is produced by multiplying a phase mask image into the original magnitude image. Both phase and magnitude sources of information complement each other and are essential for proper tissue characterization, hence, it is worthwhile to investigate the possibility of measuring the iron level on the magnitude mapping.

Thus, in the current study, we measured both phase values and R2* in each subject, and performed correlation analysis between the two methods after combining with the previously published iron concentrations [23]. We also proposed a new method using magnitude signal intensity and compared it with R2* and brain iron concentration.

## Methods

### Ethics Statement

All subjects had given written informed consent prior to the study and the protocols had been approved by the human ethics committee of the second affiliated hospital of Zhejiang university, school of medicine. All clinical investigation has been conducted according to the principles expressed in the Declaration of Helsinki.

### Participants

Twenty-four healthy adults ranging in age 45 to 88 years (mean = 68.6, SD = 11.3) participated in this study. The subjects included 13 men (54.2%) and 11 women (45.8%). Subjects with a history of neurological or psychiatric diseases, including head trauma, were carefully excluded. The subjects with evidence of focal parenchymal loss that might have resulted from infarct on T2-weighted images, and space-occupying lesions were excluded from further analysis.

### MRI protocol

All MRI studies were performed on a 3.0 T system (Signa Excite HD, General Electric Medical System, Milwaukee, USA) equipped with an 8-channel phased array head coil. Foam pads were inserted into the space between the subject's head and the MRI head coil to minimize head motion. Conventional T1- and T2-weighted images were obtained for screening of space-occupying lesions and cerebrovascular diseases in the study area, especially the basal ganglia. The whole brain was imaged. The susceptibility-weighted MR images were taken parallel to the anterior–posterior commissural line and covered the nuclei of the basal ganglia, using a three-dimensional gradient-echo sequence with the following parameters: repetition time = 58 ms; echo time = 20 ms; flip angle = 20°; matrix size = 256×256; FOV = 240×240 mm^2^; slice thickness = 2.0 mm with no gap between slices, and in-plane spatial resolution of 0.4688×0.4688 mm/pixel. Flow compensation was applied. Phase, magnitude and R2* images were acquired and all of the data were used in further analysis.

### Image processing

The raw data were transferred to a separate workstation (ADW4.4, GE) where the phase map, magnitude map and R2* map were obtained by a home built program. In this study, we used a high-pass filter with a central matrix size of 32×32 to remove background field inhomogeneities to create the corrected phase image, which were described in detail elsewhere [16], [19]. The phase values of the regions of interest (ROIs) were measured on the corrected phase images, which ranged from −π to +π. R2* data were measured on the reconstructed R2* images. These two methods were executed on the workstation.

As a new method of this study, the averaged signal intensities of the ROIs were measured on the magnitude images. To remove the individual differences of signal intensities, we then calculated the relative magnitude signal intensity (RMSI) of ROIs by dividing the mean magnitude signal intensity within each region by that of frontal white matter (FWM) to derive MRI estimates of relative iron content. These three pairs of data were used for comparison between the three methods.

### Image analysis

The ROIs included the bilateral red nucleus (RN), substantia nigra (SN), globus pallidus (GP), putamen (PU), head of caudate (CA), thalamus (TH) and frontal white matter (FWM). The basal ganglia were chosen because it has a high iron content in the brain and is easily visible on MR images [23], [24]. TH and FWM were selected to add regions of different amounts of iron.

**Figure 1:**
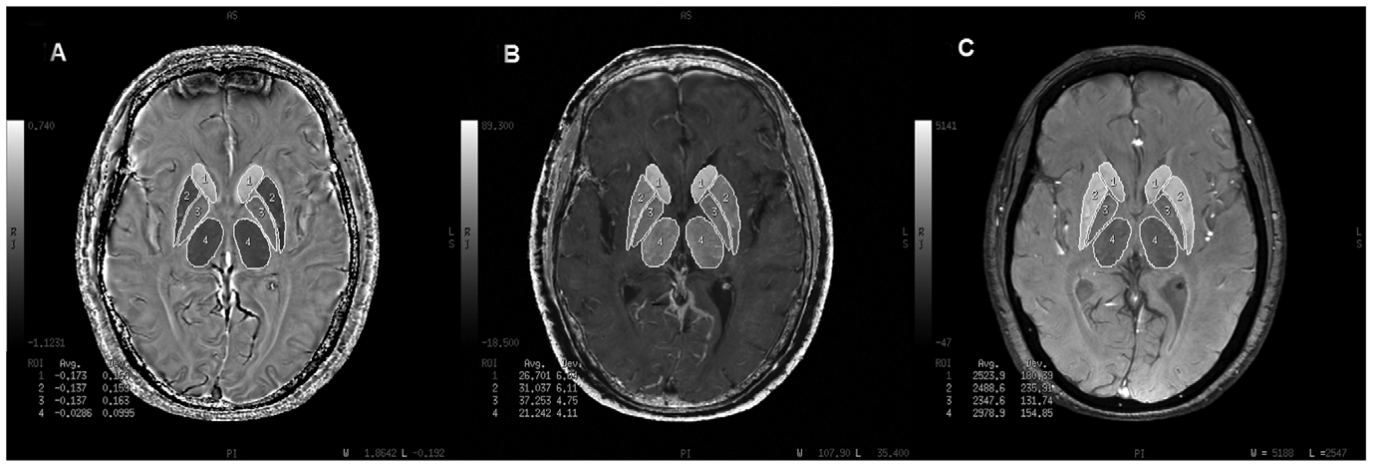
Illustration of the selected regions of interest. (A), The selected ROIs of the corrected phase images. (B), The selected ROIs of the R2* images. (C), The selected ROIs of the magnitude images. 1 = caudate; 2 = putamen; 3 = globus pallidus; 4 = thalamus.

**Figure 2:**
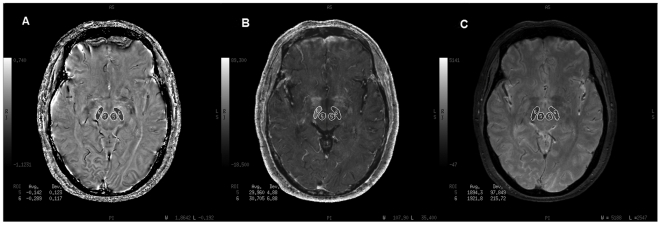
Illustration of the selected regions of interest. (A), The selected ROIs of the corrected phase images. (B), The selected ROIs of the R2* images. (C), The selected ROIs of the magnitude images. 5 = red nucleus; 6 = substantia nigra.

The ROIs of the basal ganglia and thalamus were drawn according to the anatomical structures and data for each nucleus were obtained from all visible slices and both sides, except for frontal white matter, where two consecutive slices were used. Four measurements were done on the frontal white matter with a 20-mm^2^ square ROI and then averaged to obtain a final value. ROIs on phase, R2* and magnitude images were exactly consistent by drawing at the same time on the workstation (Figure 1 & 2).

### Radiological Measurements

To demonstrate the reproducibility of the outlined ROIs of three methods, one operator (S.Y.) drew the ROIs of 10 patients twice, at an interval of 3 months apart. Another operator (J.S.) independently drew the ROIs on the same patients.

### Statistical analysis

We firstly calculated the descriptive statistics for the data of the three methods. Since the RMSI measures of ROIs were skewed towards the left of mean, we performed natural log transformations of RMSI measures before the correlation analysis. The log-transformed RMSI (log(−RMSI)) appeared to be acceptably normative in each brain region and all regions. To investigate the correlation of phase value, R2*, and log(−RMSI) in each brain region and all regions, bivariate (Pearson's) correlation analysis was used depending on the normality of the distribution, and bonferroni correction was used for comparison between multiple groups. Statistical significance was set at a probability value of ≤0.05. All statistical analyses were performed using SPSS package (14.0 for Windows).

## Results

The test-retest intraclass correlation coefficients for inter- and intra-observer agreements were 0.967 and 0.923 respectively. Because the intra- and inter-operator reliability measures were high, only one measurement (S.Y.) was used for the remainder of the ROIs.

### The values of three methods correlate with brain iron levels

**Figure 3:**
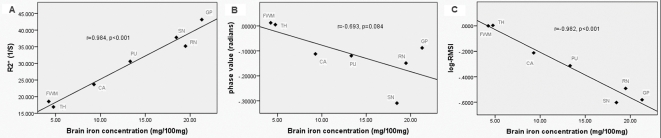
The Scatter plots between three methods and brain iron concentration as published. (A), The correlation between R2* and brain iron concentrations. (B), The correlation between phase values and brain iron concentrations. (C), The correlation between log(−RMSI) and brain iron concentration. CA = caudate; PU = putamen; GP = globus pallidus; TH = thalamus; RN = red nucleus; SN = substantia nigra; FWM = frontal white matter.

The phase values, R2*, and log(−RMSI) of each brain structure are summarized in Table 1. As a check on the validity of three measurements, we applied Pearson's correlation analysis between iron concentrations in different regions, as previously measured by biochemical methods [23], and the phase values, R2*, log(−RMSI), respectively. A strong positive correlation was found between R2* and previously published iron concentrations [23] in seven brain regions (r = 0.984, P<0.001) (Figure 3A). The results also showed a strong inverse correlation between the calculated log(−RMSI) and iron concentrations (r = −0.982, p<0.001) (Figure 3C). However, there was no significant correlation between phase value and iron concentrations (r = −0.693, p = 0.084) (Figure 3B).

**Table 1:** R2*, phase values and log(−RMSI) (mean±SD) for each brain structure.

Brain regions	R2*	phase value	Correlation&	log(−RMSI)	Correlation#
red nucleus	35.18±6.90	−0.1491±0.0709	r = −0.236, p = 0.268	−0.4919±0.1743	r = −0.593, p = 0.002
substantia nigra	37.79±8.15	−0.3093±0.1488	r = −0.723, p<0.001	−0.6028±0.1883	r = −0.521, p = 0.009
globus pallidus	43.15±7.44	−0.0880±0.0605	r = −0.111, p = 0.605	−0.5811±0.1549	r = −0.750, p<0.001
putamen	30.60±4.68	−0.1196±0.0662	r = −0.514, p = 0.010	−0.3134±0.1050	r = −0.547, p = 0.006
all measured regions	29.41±10.75	−0.1087±0.1258	r = −0.594, p<0.001	−0.3664±0.2571	r = −0.851, p<0.001

Notes.

& Correlation between phase values and R2*.

# Correlation between log(−RMSI) and R2*.

### Correlation between R2* and phase values

**Figure 4:**
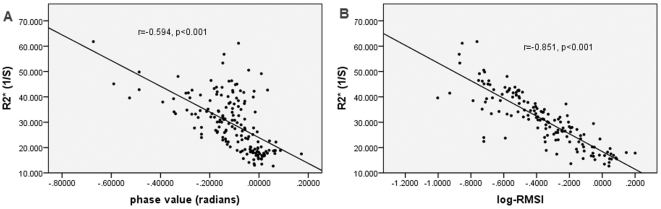
The Scatter plots between three methods in all of the measured brain regions. (A), The correlation between R2* and phase values in 168 regions. (B), The correlation between R2* and log(−RMSI) in 144 regions.

Correlation analysis of the phase values were performed with R2* of each brain region, a total number of 168 samples. The result showed a significant correlation in all regions (r = −0.594, P<0.001) (Figure 4A). To investigate the reason of no correlation between phase value and chemical brain iron content, we analyzed the correlation between phase values and R2* in four iron-rich regions (red nucleus, substantia nigra, globus pallidus and putamen). Table 1 showed correlations in substantia nigra (r = −0.723, p<0.001) and putamen (r = −0.514, p = 0.010), while no significant correlations in red nucleus (r = −0.236, p = 0.268) and globus pallidus (r = −0.111, p = 0.605).

### Correlation between R2* and log(−RMSI)

A similar analysis was performed between log(−RMSI) and R2*. The results showed a strong correlation in 144 brain regions (r = −0.851, P<0.001) (Figure 4B). Table 1 showed significant correlations in the four iron-rich regions, red nucleus (r = −0.593, p = 0.002), substantia nigra (r = −0.521, p = 0.009), globus pallidus (r = −0.750, p<0.001) and putamen (r = −0.547, p = 0.006).

## Discussion

In this study, we showed a strong linear correlation between R2* and iron concentrations reported by Hallgren and Sourander and found that R2* correlated best with iron concentrations in the human brain. This finding is consistent with recent postmortem study which indicated that R2* is more sensitive than R2 to variations in brain iron concentration [21], and thus lend further support to select R2* as a sensitive marker for iron in brain tissue.

We did not find a significant correlation between phase values and iron concentrations in seven brain regions in the current study. The iron content in GP, which is found the highest in the brain [21], [23] was lower than RN, SN, PU and even CA when detected by phase value. Similar findings was also shown in another study where iron content in GP was lower than RN and SN, and was close to the concentration in CA when phase value was used as the brain iron index [25]. These findings indicated that phase value should be used with reservation in evaluating the brain iron content. When we further compared the relationship between phase value and R2*, we found an inverse correlations in SN and PU, while no correlations in RN and GP. This implies that phase value in GP may not be an appropriate marker for in vivo brain iron content, but may be acceptable to assess iron level in SN, which plays an important role in Parkinson's disease [26]–[28].

In principle, the quantification of iron content or its susceptibility from phase images has limitations. The Fourier transform of susceptibility cannot be accurately determined in regions near the conical surfaces of magic angle [29], [30]. From the equation between iron content and phase value [31], one can deduce that the phase value changes with the difference of the magnetic susceptibility of the surrounding tissue of interest, besides the iron content. Phase value is thus related to the non-local distribution of iron and can be compromised by its dependence on object orientation.

The results of this study confirmed that the new index for evaluating the brain iron content (log(−RMSI)) based on magnitude images correlated well with R2* in all measured regions, especially in globus pallidus. They also showed a strong correlation with the reported iron concentrations throughout the brain. Since the amount of iron in the human body is approximately 30 times that of all other transition elements combined, the paramagnetic effect is mostly caused by iron. Moreover, iron not only changes the relaxation of tissue water surrounding ferritin but also introduces changes of susceptibility and microscopic field gradients. The magnitude signal intensity response for the gradient-echo sequence is given by:
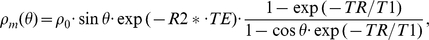
where ρ_0_ is the tissue spin density, TR is the repeat time of each data acquisition, T1 is the tissue longitudinal relaxation time, and θ is the angle by which the magnetization is tipped (usually called the flip angle). Scaling by the frontal white matter intensity may remove the signal dependence on flip angle and small T1 effect. Therefore, this signal equation explains the strong inverse correlation between log(−RMSI) and R2*. The development of the RMSI index in the region of interest compared to frontal white matter may have utility in clinical research, by eliminating the absolute value of the susceptibility changes from subject to subject. Recently, similar analysis was done based on a brain atlas-based T2 relaxation time map [32]. However, in their study, they contrasted it with FDRI and magnetic field correlation (MFC) values, but not with R2*. It might be interesting to compare our log(−RMSI) with this approach in the future study.

There are limitations to our study that need to be considered when interpreting our findings. First, independent histopathological confirmation of the three methods was not available. We compared the three indexes with the previous published iron concentrations. Therefore, it is important to point out that the correlation of brain iron content with three methods is an assumption of the approach. The methods should be further examined with both histopathological analysis and MRI scans in the same subjects. Second, all of the three indexes depend on a variety of factors other than the presence of magnetic iron particles, such as regional tissue characteristics and imaging parameters include voxel size, field strength and echo time. This limitation may also find in other techniques, such as FDRI technique that further requires the use of two different field-strength MRI instruments. Third, the calcification in the brain tissue may be confused with iron deposits in these measurements. The new quantitative susceptibility mapping technique by solving the field to susceptibility source inverse problem is promising to address the second and third limitations [30].

In conclusion, we analyzed the relationship between three methods used for assessing the brain iron content, including phase value, R2* and log(−RMSI), and we found that R2* and log(−RMSI) are both good markers, while phase value has limitations. Future prospective studies using more advanced MRI techniques and postmortem studies to confirm our findings.
